# Late presentation for HIV care in Southwest Ethiopia in 2003–2015: prevalence, trend, outcomes and risk factors

**DOI:** 10.1186/s12879-018-2971-6

**Published:** 2018-01-30

**Authors:** Hailay Abrha Gesesew, Paul Ward, Kifle Woldemichael, Lillian Mwanri

**Affiliations:** 1 Public Health, Flinders University, Adelaide, Australia; 2 Epidemiology, Jimma University, Jimma, Ethiopia

**Keywords:** Trend, Outcomes, Late presentation, Retrospective cohort, Ethiopia

## Abstract

**Background:**

Early presentation for HIV care is vital as an initial tread in the UNAIDS 90–90–90 targets. However, late presentation for HIV care (LP) challenges achieving the targets. This study assessed the prevalence, trends, outcomes and risk factorsfor LP.

**Methods:**

A 12 year retrospective cohort study was conducted using electronic medical records extracted from an antiretroviral therapy (ART) clinic at Jimma University Teaching Hospital. LP for children refers to moderate or severe immune-suppression, or WHO clinical stage 3 or 4 at the time of first presentation to the ART clinics. LP for adults refers to CD4 lymphocyte count of < 200 cells/ μl and < 350 cells/μl irrespective of clinical staging, or WHO clinical stage 3 or 4 irrespective of CD4 count at the time of first presentation to the ART clinics. Binary logistic regression was used to identify factors that were associated with LP, and missing data were handled using multiple imputations.

**Results:**

Three hundred ninety-nine children and 4900 adults were enrolled in ART care between 2003 and 15. The prevalence of LP was 57% in children and 66.7% in adults with an overall prevalence of 65.5%, and the 10-year analysis of LP showed upward trends. 57% of dead children, 32% of discontinued children, and 97% of children with immunological failure were late presenters for HIV care. Similarly, 65% of dead adults, 65% of discontinued adults, and 79% of adults with immunological failure presented late for the care. Age between 25- < 50 years (AOR = 0.4,95% CI:0.3–0.6) and 50+ years (AOR = 0.4,95% CI:0.2–0.6), being female (AOR = 1.2, 95% CI: 1.03–1.5), having Tb/HIV co-infection (AOR = 1.6, 95% CI: 1.09–2.1), having no previous history of HIV testing (AOR = 1.2, 95% CI: 1.1–1.4), and HIV care enrollment period in 2012 and after (AOR = 0.8, 95% CI: 0.7–0.9) were the factors associated with LP for Adults. For children, none of the factors were associated with LP.

**Conclusions:**

The prevalence of LP was high in both adults and children. The majority of both children and adults who presented late for HIV care had died and developed immunological failure. Effective programs should be designed and implemented to tackle the gap in timely HIV care engagement.

## Background

The Joint United Nations Program on HIV and AIDS (UNAIDS) has developed a 90–90–90 treatment target framework in order to end AIDS globally which aims at 90% of people living with HIV knowing their HIV status, 90% of HIV diagnosed patients receiving sustained treatment, and 90% of those on HIV treatment achieving viral suppression [1]. While diagnosing HIV infection is vital as the initial tread in the 90–90­90 targets, diagnosis per se is no longer sufficient [2]. Early diagnosis and access to treatment helps people with HIV to timely get and appropriately use HIV treatment [3] that further reduces the virus load and risk of morbidity and mortality. Nevertheless, late presentation for HIV care (LP) has been recognized as an impediment to meet the above mentioned UNAIDS targets.

LP is the result of being late in HIV diagnosis and/or late in linking with or in accessing HIV care [4]. The definition of LP is disparate and is contextualized using the threshold for ART eligibility [5]. To date, numerous definitions have been used including: i) when the baseline CD4 count is < 200 or < 350 cells/μl and/or with an AIDS defining disease [3, 6, 7], ii) when AIDS defining conditions are diagnosed either before or during the period to an HIV diagnosis [8], iii) when AIDS defining conditions are diagnosed in the subsequent 6 months period to an HIV diagnosis [9], or iv) when AIDS defining conditions are diagnosed 12 months period to an HIV diagnosis [10].

LP is associated with increased risk of HIV transmission [11], ART drug resistance [11], and health care expenses [12]. Additionally, LP has been acknowledged as a challenge for the achievement of the ambitious UNAIDS 90–90-90 targets [13, 14]. For the first 90, high magnitude of late HIV diagnosis reflects that there are a number of people who did not know their HIV status. For the second 90, LP results in poor health outcomes and this interrupts the sustainable uptake of the treatment [13]. Furthermore, LP significantly contributes for pre-ART deaths and, this in turn, reduces the number of HIV diagnosed patients on ART [15]. For example, a study from South Africa reported that ART initiation at the time of first presentation to ART clinic boosted treatment uptake by 36% [16]. For the third 90, LP lowers the number of CD4 cells and increases the number of viral counts, and this causes clinical, immunological or virological failure [14, 17]. Previous studies have shown that late diagnosis appeared to be the main reason for virological failure, and ART initiation at first visit increased viral suppression by 26% [16].

LP has been reported to be a significant problem across the globe. In Europe, overall prevalence of 15–66% has been reported [18, 19]. The magnitude of LP in Asia was very significant (72–83.3%) [20], and in Africa, the overall prevalence has been reported to be between 35 and 65% [21, 22]. Nonetheless, heterogeneity in its measurement limited direct comparisons [23]. In Ethiopia in 2015, there were 39,140 new infections, 768, 040 people living with HIV and 28, 650 HIV/ADS deaths [24]. Universal access to ART in the country was launched in 2005 [25], and to date, the coverage of ART—the percentage of people on ART among those in need of treatment [26]— is 52% [24]. However, the status of timely presentation for HIV care is yet to be assessed. One cross-sectional study from northern part of the country has reported a LP prevalence of 68.8% [27].

Demographic, behavioural and clinical factors contributed for LP [6, 20, 28, 29]. For instance, being female, older age, rural dwellers, alcohol users, ‘*Khat*’ chewers, cigarette smokers, being diagnosed with sever co-morbidities, perceiving HIV related stigma, having contact with commercial sex workers and being exposed to risky sexual behavior were the factors associated with LP [6, 20, 28, 29]. In Ethiopia, other studies have assessed factors affecting LP [6, 27, 29], and all except one were from the northern part of the country.

However, it is well known that the southwestern part of the nation has different cultural and socioeconomic characteristics. It also has the highest HIV prevalence (6.5%) in the country [30] and may have different LP factors which need to be understood to address HIV in these settings. In addition, for patients who started ART, no study has been conducted to assess the outcome and trends of LP. The prevalence of LP among children has also not been determined. Given the above gaps, and the clinical and public health importance of early HIV diagnosis on timely ART commencement, it is imperative to comprehend the LP situation and recommend effective programs that facilitate early presentation for HIV care in Ethiopia. Furthermore, addressing LP may have a substantial contribution for SDG-3 to have good health and wellbeing, and particularly for SDG-3.3 to end HIV epidemics by 2030. This paper examines the prevalence, trend, outcomes and risk factors of LP among children and adults enrolled for ART in Jimma University Teaching Hospital (JUTH), Southwest Ethiopia.

## Methods

### Study design, setting and participants

A retrospective cohort study was undertaken using data extracted from the ART clinic at JUTH using patient records from June 2003 to March 2015. Details of the study setting has been described elsewhere [31]. The study population was all HIV patients enrolled for ART care in JUTH.

### Data source and procedures

The data were extracted from JUTH electronic medical records (EMR) system called comprehensive care center patient application database (C-PAD) that was in place since 2007. HIV care providers record patient clinical and non-clinical information on paper form, which is then entered into the EMR by data clerks. Two data clerks perform the data entry process to ensure completeness. The International Center for AIDS Care and Support (ICAP) at Colombia University was also delivering technical assistance on the electronic patient level data management, and has been conducting random check up of data completeness. This ensures the accuracy and reliability of the EMR data. Weekly patient summary generated from the EMR system helps to flag patients with conditions that seek follow-up. If baseline CD4 and WHO clinical staging were not recorded, records would be excluded.

### Study variables and definitions

The dependent variable was the time when a patient presented for HIV care and was dichotomized as late or early. We defined LP for adults when the baseline CD4 cells count is < 200 cells/μl or < 350 cells/μl (pre-and post-revision of national ART guideline) irrespective of WHO clinical staging, or WHO clinical stage 3 or 4 irrespective of CD4 count at the time of first presentation to an ART clinic [3, 6]. Early presentation for HIV care (EP) is the opposite of LP. LP and EP for children are defined in Table 1. The independent variables included age, sex, marital status, educational status, religion, Tb/HIV co-infection, baseline functional status, history of HIV testing, and HIV care enrollment period. History of previous HIV testing refers to testing (one or more times) for HIV before diagnosis. HIV care enrollment period was dichotomized as enrolled for HIV care in 2003–11, and 2012 and after.

**Table 1:** Measurements for late presentation for HIV care (LP)

Adults	Late presentation for HIV care [6, 35]^a^		
	Enrolled in 2003–11	Enrolled in 2012–15	
	CD4 lymphocyte count of < 200 cells/ μl irrespective of WHO clinical stage at the time of first presentation to the HIV care	CD4 lymphocyte count of < 350 cells/ μl irrespective of WHO clinical stage at the time of first presentation to the HIV care	
	WHO clinical stage 3 or 4 irrespective of CD4 count at the time of first presentation to the HIV care ^b^	WHO clinical stage 3 or 4 irrespective of CD4 count at the time of first presentation to the HIV care	
Children^c^	Late presentation for HIV care [70]		
		Moderate immune-suppression (damage) if CD4 count between	Severe immune-suppression (damage) if CD4 count between
	0–12 months	750–1500 cells/ μl	< 750 cells/ μl
	1–5 years	500–1000 cells/ μl	< 500 cells/ μl
	≥ 6 years	200–500 cells/ μl (enrolled in 2003–2011)	< 200 cells/ μl (enrolled in 2003–11)
	≥ 6 years	350–500 cells/ μl (enrolled in 2012–2015)	< 350 cells/ μl (enrolled in 2012–2015)

ART: antiretroviral therapy; CD4: cluster for differentiation 4; WHO: World Health Organization; Tb: Tuberculosis; PCP: pneumocystis carinii (juvenii) pneumonia

^a^The definition for LP among Tb/HIV co-infected population was only based on the CD4 criteria [4]

^b^WHO clinical Stage 3was defined if one of the following is present in an HIV diagnosed patient: weight loss of > 10% body weight, chronic diarrhea for > 1 month, fever for > 1 month, oral candidiasis, oral hairy leukoplakia, or pulmonary Tb within the previous year, or severe bacterial infections; WHO clinical Stage 4 was defined if one of the following is present in an HIV diagnosed patient: HIV wasting syndrome, PCP, toxoplasmosis of the brain, cryptosporidiosis or isosporiasis with diarrhea for > 1 month, cytomegalovirus disease of an organ other than liver, spleen or lymph node, herpes simplex virus infection, progressive multifocal leukoencephalopathy, candidiasis, extra-pulmonary Tb, lymphoma, kaposi’s sarcoma, HIV encephalopathy

^c^LP is also defined if WHO clinical stage 3 or 4 at first visit to the ART clinics

Possible outcomes of LP were ART discontinuation, immunological failure and mortality. ART discontinuation is attributed to lost to follow up (LTFU), defaulting and stopping medication while remaining in care. LTFU was if patients had been on ART treatment and had missed at least three clinical appointments but had not yet been classified as “dead” or “transferred out” (TO). Defaulting was if patients had been on ART treatment and had missed less than three clinical appointments but had not yet been classified as “dead” or “TO”. Stopping medication was definedwhen patients had stopped treatment due to any reason while they have remained in care. TO is the official transferring of the patient to another ART clinic within or outside a catchment area. Immunological failure was defined based on the definitions provided by WHO [32]. Mortality (all cause mortality) is the death of people on ART in the reporting period. Table 1 reports the measurements of LP.

### Statistical analyses

Ten year trends (data for years 2003 and 2015 were excluded since their number of months were incomplete) for LP was described by line graphs, and best-fit equation for the trend line was developed. We described the percentage of LP by ART discontinuation, immunological failure and mortality to show the outcomes of LP. The differences between LP and its outcomes were checked using Chi square tests. We used binary logistic regression to identify factors that were associated with LP. Multi-collinearity was checked using variance inflation factor and none was found. In addition, we checked for potential two-way interactions and none was found.

Missing data was treated using multiple imputations (*n* = 5) assuming missing at random (MAR) pattern [33] and the model was reported with pooled imputed values [34]. We developed an imputation model for adults, however; for children, we did not decide to do the multiple imputations analysis since the missing values for the great majority of the variables were not significant (below 5%). Bivariate logistic regression analysis was conducted to determine the presence of crude association and nominate the candidate variables (*P* < 0.25 was considered) to multiple logistic regression. *P*-value ≤ 0.05 was considered as a cutoff value for statistical significance in the final multiple logistic regression model. We applied Hosmer and Lemeshow test to check goodness of fit of the final model and was found fit (P–value = 0.17). We reported odds ratio and 95% confidence interval to summarize the data. We used Statistical Package for the Social Sciences (SPSS) version 22.0 for all data analyses.

## Results

Of the 8172 patients enrolled for HIV care between 21 June 2003 and 15 March 2015, 5299 (64.8%) patients on ART, the study population for the study, were included. Of the total patients enrolled for ART, 4900 (92.5%) were adults and 399 (7.5%) were children.

Table 2 demonstrates the characteristics of HIV patients on ART. Among the children, 58.1% were aged 5–15 years, 52.4% were males, and 73.9% were Christians. The majority of the children (79.4%) had moderate or severe immune-suppression, and half of the children had baseline WHO clinical stage 3 or 4. A total of 114 (28.6%) children had Tb/HIV co-infection. The median time on ART and estimated survival time, respectively, was 40 and 104.2 (99.8–108.5) months. Among adults, three fifth (59.8%) were females, about half (48.7%) were married and 39% had primary school education. Two fifth (41.6%) of adults had no history of HIV testing. A significant number of adults (73.6%) had baseline CD4 count below 200 cells/mm^3^, and 54.3% had WHO clinical stage 3 or 4. Over a quarter of adults (27.9%) had Tb/HIV co-infection. Twenty nine (0.9%) adults changed ART regimen during the course of the period. The median time on ART was 49 months, and the median estimated survival time was 121.9 (95%CI: 120.3–123.5) months.

**Table 2:** Clinical & non-clinical characteristics of HIV infected people enrolled in ART care in Southwest Ethiopia from 2003 to 2015

Variable		Children (*N* = 399)	Adult (*N* = 4900)
		*N* (%)	*N* (%)
Age in years	< 1	21 (5.3)	–
	1- < 5	146 (36.6)	–
	5- < 15	232 (58.1)	–
	15- < 25	–	711 (14.5)
	25- < 50	–	3937 (80.3)
	50+	–	252 (5.2)
	Median (range) age in years	6 (< 1–14)	30 (15–81)
ART follow up time in months, median (range)		40 (0–116)	49 (0–137)
Estimated survival time in months, median (95%CI)		104.2 (99.8–108.5)	121.9 (120.3–123.5)
Sex	Male	209 (52.4)	1971 (40.2)
	Female	190 (47.6)	2929 (59.8)
Marital status	Never married	–	897 (18.3)
	Married	–	2094 (42.7)
	Separated/divorced/widowed	–	1311 (26.8)
	Missing	–	598 (12.2)
Education	No education	–	945 (19.3)
	Primary	–	1687 (34.4)
	Secondary and above	–	1685 (34.4)
	Missing	–	583 (11.9)
Religion	Muslim	47 (11.8)	1402 (28.6)
	Christian^b^	133 (33.3)	2893 (59)
	Missing	219 (54.9)	605 (12.3)
Baseline WHO classification	1 or 2	108 (27.1)	1355 (27.7)
	3 or 4	110 (27.6)	1608 (32.8)
	Missing	181 (45.3)	1937 (39.5)
Baseline CD4 count category	No damage	72 (20.6)	–
	Moderate or severe damage	277 (79.4)	–
	Median (range) CD4 count	282 (0–2250)	–
Baseline CD4 count (cells/mm3)	< 200	156 (39.1)	3275 (66.8)
	≥ 200	193 (48.4)	1174 (24)
	Missing	50 (12.5)	451 (9.2)
	Median (range)	282 (0–2250)	156 (0–1313)
History of Tb/HIV co-infection	No	285 (71.4)	3533 (72.1)
	Yes	114 (28.6)	1367 (27.9)
ARV adherence	Good	319 (79.9)	4064 (82.9)
	Fair or poor	80 (20.1)	836 (17.1)
Cotrimoxazole adherence	Good	315 (78.9)	4119 (84)
	Fair or poor	84 (21.1)	762 (15.6)
	Missing	–	19 (0.4)
History of HIV testing	Yes	399 (100)	2860 (58.4)
	No	0 (0)	2040 (41.6)
ART shift	No	214 (97.7)	3190 (65.1)
	Yes	5 (2.3)	29 (0.6)
	Missing	180 (45.1)	1681 (34.3)
Baseline functional status	Appropriate	170 (42.6)	–
	Delay or regression	229 (57.4)	–
Baseline functional status	Work or Ambulatory	–	3064 (62.5)
	Bedridden	–	1437 (29.3)
	Missing	–	399 (8.1)
Timing to HIV care presentation	Early	162 (40.6)	894 (18.2)
	Late	215 (53.9)	1788 (36.5)
	Missing	22 (5.5)	2218 (45.3)
Baseline CD4 count in cells/mm3 by HIV care enrollment period (median (range))	enrolled in 2003–11	273 (0–2000)	119 (0–1641)
	enrolled in 2012 and after	368 (3–2247)	178 (0–1638)

^a^Orthodox, Catholic, Protestant

### Prevalence, trend and outcomes of LP

**Fig. 1:**
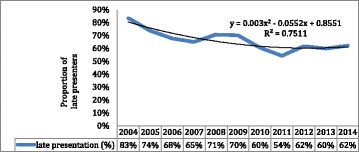
Trends in the percentage distribution of late presentation for HIV care among HIV infected people on ART, Southwest Ethiopia, 2004-2014

The overall prevalence of LP was 65.5%, and females accounted for the majority (64.3%).In total, 215 children (57%) and 1788 adults (66.7%) were late presenters. In the period between 2004 and 2014 the percentage of LP decreased from 83% to 62%. Fig. 1 shows the trend in LP among HIV infected people on ART.

Table 3 reports the outcomes of LP among HIV infected children and adults enrolled in HIV care. Of the children, 57.1% of those who died, 32.3% of those who discontinued care, and 96.9% of those who had immunologic failure were late presenters for HIV care. Of adults, 64.7% of those who died, 65.3% of those who discontinued care, and 78.7% of those who had immunologic failure presented late for the HIV care. The chi-square analysis showed that the difference between LP and, immunologic failure and discontinuation for children was statistically significant. For adults, LP was not statistically different with mortality and ART discontinuation.

**Table 3:** Outcomes of late presentation for HIV care among HIV infected children and adults enrolled in HIV care in southwest Ethiopia, 2016

Age	Variable	Mortality		Discontinuation		Immunological status	
		Alive, n(%)	Death, n(%)	Retained, n(%)	Discontinued, n(%)	IS, n(%)	IF, n(%)
Children	EP	64 (44.8)	3 (42.9)	64 (44.8%)	21 (67.7)	40 (25.3)	1 (3.1)
	LP	79 (55.2)	4 (57.1)	79 (55.2%)	10 (32.3)	118 (74.7)	31 (96.9)
	Total	143 (100)	7 (100)	143 (100)	31 (100)	158 (100)	32 (100)
	*P*-value (of X^2^)	0.921		0.020		0.005	
Adults	EP	459 (33.1)	65 (35.3)	459 (33.1)	184 (34.7)	682 (36.5)	99 (21.3)
	LP	927 (66.9)	119 (64.7)	927 (66.9)	347 (65.3)	1187 (63.5)	365 (78.7)
	Total	1386 (100)	184 (100)	1386 (100)	531 (100)	1869 (100)	464 (100)
	*P*-value (of X^2^)	0.550		0.524		< 0.001	

*EP* early presentation for HIV care, *LP* late presentation for HIV care presentation, *IF* immunologic failure, *IS* Immunologic success, *X*^*2*^ Chi-square

### Risk factors for LP

Table 4 presents the multiple logistic regression analysis of factors for LP obtained from the complete case analyses. Predictors of LP among adults included being older adult, female, having Tb/HIV co-infection, having no previous history of HIV testing and HIV care enrollment period in 2012 and after. Older adults aged between 25- < 50 years (AOR = 0.4, 95% CI: 0.3–0.6) and 50+ years (AOR = 0.4, 95% CI: 0.2–0.6) were 60% each less likely to present late for HIV care compared to those aged between 15 and 25 years. Females were 20% high likely (AOR = 1.2, 95% CI: 1.03–1.5) to present late for HIV care than their comparator. HIV patients with Tb/HIV co-infection were about 2 times at risk of LP (AOR = 1.6, 95% CI: 1.09–2.1) than HIV patients alone. In addition, while having no previous history of HIV testing (AOR = 1.2, 95%CI: 1.1–1.4) was a risk factor for LP, HIV care enrollment period in 2012 and after (AOR = 0.8, 95% CI: 0.7–0.9) was a protective factor for LP. No statistically significant predictor was found for LP among children.

**Table 4:** Logistic regression findings of factors linked with late presentation for HIV care in HIV infected people, JUTH, Southwest Ethiopia, 2016

Variable		Children		Adults		Children		Adults		
		Time at presentation for HIV care		Time at presentation for HIV care						
		Early, n (%)	Late, n (%)	Early, n (%)	Late, n (%)	COR (95%CI)	AOR (95%CI)	COR (95%CI)	AOR (95%CI): Complete cases	AOR (95%CI): Multiple imputations
Age	< 1	7 (36.8)	12 (63.2)	–	–	1	1	–	–	–
	1- < 5	56 (42.1)	77 (57.9)	–	–	0.8 (0.3–2.2)	0.5 (0.1–2.6)	–	–	–
	5- < 15	99 (44)	126 (56)	–	–	0.7 (0.3–1.9)	0.4 (0.1–2.2)	–	–	–
	15- < 25	–	–	96 (73.8)	340 (26.2)	–	–	1	1	1
	25- < 50	–	–	739 (35.2)	1362 (64.8)	–	–	0.5 (0.4–0.7)^a^	0.4 (0.3–0.6)^a^	0.5 (0.4–0.7)^a^
	50+	–	–	59 (40.7)	86 (59.3)	–		0.4 (0.3–0.7)^a^	0.4 (0.2–0.6)^a^	0.4 (0.3–0.6)^a^
Sex	Male	91 (46.2)	106 (53.8)	359 (37.1)	609 (62.9)	1		1	1	1
	Female	71 (39.4)	109 (60.4)	535 (31.2)	1179 (68.8)	1.4 (0.9–1.9)		1.3 (1.1–1.5)^a^	1.2 (1.03–1.5)^a^	1.2 (1.003–1.4)^a^
Marital status	Never married	–	–	151 (30.2)	349 (69.8)	–	–	1	1	1
	Married	–	–	391 (33.6)	772 (66.4)	–	–	0.9 (0.7–1.1)	0.8 (0.7–1.07)	0.8 (0.7–1.05)
	Separated or divorced or widowed	–	–	238 (31.9)	509 (68.1)	–	–	0.9 (0.7–1.2)	0.9 (0.6–1.1)	0.9 (0.7–1.2)
Educational status	No education	–	–	149 (32.5)	309 (67.5)	–	–	1		
	Primary	–	–	320 (34.8)	599 (65.2)	–	–	0.9 (0.7–1.2)		
	Secondary and above	–	–	313 (30.2)	722 (69.8)	–	–	1.1 (0.9–1.4)		
Religion	Muslim	16 (37.2)	27 (62.8)	245 (33.1)	496 (66.9)	1		1		1
	Christian ^b^	52 (40.9)	75 (59.1)	535 (32.3)	1123 (67.7)	0.9 (0.4–1.7)	0.9 (0.4–1.9)	1.1 (0.9–1.3)		1.02 (0.9–1.2)
Tb/HIV co-infection	No	120 (45.5)	144 (54.5)	656 (34.5)	1244 (65.5)	1	1	1	1	1
	Yes	42 (37.2)	71 (62.8)	238 (30.4)	544 (69.6)	1.4 (0.9–2.2)	1.3 (0.7–2.7)	1.2 (1.01–1.4)^a^	1.6 (1.09–2.1)^a^	1.2 (1.00–1.4)^a^
Baseline functional status	Appropriate	66 (42.6)	89 (57.4)	–	–	1	1	–	–	–
	Delay or regression	96 (43.2)	126 (56.8)	–	–	1.03 (0.7–1.6)	1.1 (0.5–1.9)	–	–	–
Baseline functional status	Working or ambulatory	–	–	542 (32)	1150 (68)	–	–	1	1	1
	Bedridden	–	–	293 (36.9)	500 (63.1)	–	–	0.8 (0.7–1.1)	0.8 (0.6–1.002)	0.8 (0.7–1.001)
History of previous HIV testing	Yes	162 (43)	215 (57)	529 (34.4)	1008 65.6)	–	–	1	1	1
	No	0	0	365 (31.9)	780 (68.1)	–	–	1.1 (0.9–1.3)	1.2 (1.1–1.4)^a^	1.1 (1.00–1.3)^a^
HIV care enrollment period	enrolled in 2003–11	128 (42.2)	175 (57.8)	698 (32.1)	1478 (67.9)	1		1	1	1
	enrolled in 2012 and after	34 (45.9)	40 (54.1)	196 (38.7)	310 (61.3)	0.9 (0.5–1.4)		0.7 (0.6–0.9)^a^	0.8 (0.7–0.9)^a^	0.7 (0.5–0.9)^a^

*COR* crude odds ratio, *AOR* adjusted odds ratio, *CI* confidence interval, *Tb/HIV* tuberculosis/HIV, *ART* antiretroviral therapy,^a^statistically significant at *P*-value = 0.05; ^b^orthodox, protestant, catholic

### Multiple imputations (MI)

To handle the missing data, we applied MI using five imputed data sets. We have presented the results from MI and complete case analyses in Table 4. In estimating factors associated with LP among adults, results were similar in both MI and complete case analyses except for variables Tb/HIV co-infection and previous history of HIV testing were marginally statistically significant in the MI analysis.

## Discussion

LP has been described as a sizable obstacle to attaining the UNAIDS 90–90-90 and 95–95-95 targets [7, 14]. This study has shed light on the general problems of late HIV care—magnitude, trend, outcomes and its risk factors. In the current study, the overall prevalence of LP was considerably high (65.5%). Furthermore, the trends of LP had shown persistently elevated prevalence (between 54% and 83%) although a lessening pattern was observed. This finding is consistent with another finding conducted in the country [27]. The prevalence of LP in the current study (65.5%) was lower (72–83.3%) than the prevalence from studies conducted in Asia [20], but higher than the findings from other studies in Africa that reported between 35 and 65% [21, 22]. This implies that LP in Ethiopia is still highly prevalent even after the introduction of universal ART.

The high and persistent LP prevalence may be due to: i) lack of information [35], ii) persistently high level of HIV related stigma [3, 27], iii) low HIV risk perception [27, 35] especially among high risk groups [36], iv) use of traditional treatment [27], v) poor integration between modern medicine and traditional healers, and vi) phasing out of international funding agencies [37]. Additionally, it could also be attributed to poor access to HIV services [37, 38]. For example, only 79% of the total health facilities in Ethiopia deliver HIV counseling and testing services [39]. Primary Health Care principles and scholars describe characteristics of accessible health systems to be approachable, acceptable, available, affordable and appropriate for the target population [40, 41], and thus raising a question whether HIV services are accessible to all HIV patients in Ethiopia. As such, LP issues should be given top priority if Ethiopia is to meet the UNAIDS targets.

Several strategies including the use of technology have been recommended to reduce LP prevalence in developing countries. For example, in collaboration with UNICEF, Amukele and colleagues successfully piloted unmanned aerial systems (drones) for transporting laboratory specimens to reduce late infant diagnosis in Malawi [42]. Other programs such as using mobile text messages [14], home [43] and community-based HIV testing [44, 45] have also been recommended to meet the HIV diagnosis target. Furthermore, encouraging repeat HIV testing [46, 47], HIV testing services delivery by lay workers [48], and self-testing [49] can tackle the substantial gap in HIV diagnosis in low-income countries like Ethiopia. The mandatory HIV testing strategy that has been implemented in China since 2005 for testing at-risk groups such as drug users, inmates, and commercial sex workers along with their clients was found to be an effective strategy to heighten early HIV diagnosis [7] that Ethiopia could consider. Interventions such as lay counselor home visits [50], home visits by peer supporters [51] and informational brochure provision [52] were also important programs in linking patients to ART clinics timely after HIV testing. In addition, the application of rapid or point of care CD4 count technology has shown to enhance the number of eligible patients for ART whereby the frequency of appointments is reduced, and early ART initiation is increased [53].

Compared to the early presenters, great majority of late presenters had died in the current study, and this is similar with findings from other studies conducted elsewhere [54, 55]. It is plausible therefore to argue that late presentation leads to a greater risk of: rapid progression to advanced AIDS stage, compromised immune response, poor treatment response, and finally death [3]. Similarly, consistent with findings of other studies [56, 57], the majority of adults who presented late for care had discontinued and developed immunologic failure. This could be highly possible since late presenters progress easily to advanced AIDS stage, a stage characterized by marked CD4 reduction, multiple comorbidities and poor overall functional status [58, 59]. Subsequently, this leads to poor immune recovery even after treatment initiation, and increases the likelihood of ART toxicity that deters patients to take the treatment regularly [60]. The prevalence of LP among adults was higher than children. This might largely be due to the ‘opt out’ screening programs for pregnant women and delivery of HIV care (testing and treatment) to children born to affected mothers timely [61].

Adult late presenters were more likely to be younger, females, Tb/HIV co-infected, with no history of HIV testing and enrolled to HIV care in 2011 and before. Unlike other studies in Africa [62, 63], older adults were less likely to delay for HIV care than their younger counterparts. We found the finding surprising. HIV disease progresses with time, and it would be expected, that individuals diagnosed with HIV at a higher age would also have advanced disease progression (lower CD4 cell count) because they, on average, had a longer time span between time of infection and time of diagnosis. However, the presence of high HIV related stigma among young adults [64] hampers HIV testing and may be linked to delays in seeking HIV care. In addition, it is also possible that older adults assume a caring responsibility for their family and might realise the need to access HIV care service consistently increase their longevity and to achieve the self-imposed caring responsibility. Unlike in some others [56, 63], females were more likely than males to delay for HIV care. This might be because females have low understanding and comprehensive HIV care knowledge [3]. The high probability of females for LP might also be explained by the fact that 62% of females in care did not have a previous history of HIV testing. Females are also known to use traditional healers more than males, which may lead to commencing the HIV care late [3, 35].HIV related stigma is higher among females than males [65]. It is also known that the health seeking behaviours of females in urban and rural southwest Ethiopia are lower compared to males [66].

The association between Tb/HIV co-infection with increased LP replicate findings from other studies [4]. It has been stated that Tb is inextricably linked with HIV, causes a synergistic combination of illness with HIV, facilitates the progression of HIV disease to advanced stage, and thereby deters patients from linking to care timely [31]. Furthermore, focus has to be given for Tb/HIV co-infection, as Tb remains the highest mortality risk among HIV infected patients [13]. The absence of previous history of HIV testing in association with LP could be linked with poor awareness of the care [3], less access to HIV testing and/or ART clinic [39], high HIV related stigma [67], fear of diagnosis [68] and feeling of wellbeing [69].HIV patients who were enrolled for HIV care in 2012 and after were less likely to present late for HIV care as compared to those enrolled in 2011 and before. This may be attributed to: i) improving awareness to HIV care; ii) improving access and availability to HIV care; and iii) reducing perceived HIV related stigma.

The study has the following limitations. Firstly, data were collected from JUTH, a referral hospital that also receives referrals of patients with advanced stage. Hence, these study participants are not necessarily representative of HIV patients who attend their follow up in health centers or lower health care setups. Secondly, we did not assess the annual proportions of LP across HIV testing strategies (voluntarily counseling and testing, provider initiated HIV testing and counseling (PITC), Outreach or ‘opt out). Previous studies have shown that PITC was not found more effective program for early HIV diagnosis than targeted HIV counseling [7]. Thirdly, the use of conservative definitions for LP is not able to differentiate whether the late presentation is before diagnosis, between diagnosis and first entry to care, and between first entry to care and ART initiation. A gold standard definition for LP among general HIV positive population and special groups such as HIV positive mothers and Tb/HIV co-infected patients for low-income countries is yet to be established. Fourthly, we found no statistically significant predictor for LP among children, and this could be due to small sample size. Finally, the presence of incomplete data may bias the precision of estimates.

However, even with the aforementioned limitations, the study sheds light and underpins the high prevalence of LP. Furthermore, the research assessed outcomes and risk factors for LP across ages, recommended effective programs and benchmarking strategies to tackle LP, and further achieve the ambitious UNAIDS targets**.**

## Conclusions

In summary, three of five HIV infected people presented late for HIV care, and the annual proportions of LP persistently remained high across the 10 year period. The majority of HIV infected children and adults who presented late for care had discontinued, transferred out and developed immunologic failure. Late presenters (adults) were more likely to be younger, females, Tb/HIV co-infected, no history of HIV testing before diagnosis, and enrolled to HIV care before 2012. A large sample size should be considered to assess factors influencing for LP among children. Prioritizing the aforementioned risk factors, tremendous efforts are necessary to curb LP and further achieve the UNAIDS goal. Some of the strategies and programs that help to decline LP are use of innovative technologies, home and community based HIV testing, encouraging repeat and self-HIV testing, mandatory HIV testing and effective linking strategies to care after HIV diagnosis. HIV related stigma should also contextually be tackled since it continues to be a lingering issue among HIV infected people.

## Abbreviations

AOR: Adjusted odds ratio
ART: Antiretroviral therapy
CD4: Cluster of differentiation 4
CI: Confidence interval
COR: Crude odds ratio
HIV: Human immunodeficiency virus
JUTH: Jimma University Teaching Hospital
LP: Late presentation for HIV care
PITC: Provider initiated HIV testing and counseling
Tb: Tuberculosis
UNAIDS: The Joint United Nations Program on HIV and AIDS
WHO: World Health Organization
